# Ethanol at Low Concentration Attenuates Diabetes Induced Lung Injury in Rats Model

**DOI:** 10.1155/2014/107152

**Published:** 2014-06-12

**Authors:** Jun-Feng Hu, Guan-Jun Zhang, Lei Wang, Pin-Fang Kang, Jun Li, Hong-Ju Wang, Qin Gao, Yu-Qing Chen

**Affiliations:** ^1^Department of Respiratory Disease, The First Affiliated Hospital of Bengbu Medical College, Bengbu, Anhui 233004, China; ^2^Department of Physiology, Bengbu Medical College, 2600 Dong Hai Avenue, Bengbu 233030, China; ^3^Department of Cardiovascular Disease, The First Affiliated Hospital of Bengbu Medical College, Bengbu, Anhui 233004, China; ^4^Dongzhimen Hospital, Beijing University of Chinese Medicine, Beijing 100700, China

## Abstract

To observe the changes of lung injury when diabetic rats were treated with low concentration of ethanol (EtOH) and analyze the related mechanisms, male Sprague-Dawley (SD) rats were divided into control, diabetic (DM), and EtOH+DM groups. Diabetic rat was mimicked by injection of streptozotocin intraperitoneally. Fasting blood glucose (FBG) level, lung weight (LW), body weight (BW), and LW/BW were measured. The changes of lung tissue and Type II alveolar cell were detected. Pulmonary malondialdehyde (MDA) content and superoxide dismutase (SOD) activity were measured; meanwhile, ALDH2 mRNA and protein expressions were detected by RT-PCR and western blotting, respectively. Compared with control group, in DM group, SOD activity was decreased; FBG level, LW/BW, MDA content, ALDH2 mRNA, and protein expressions were decreased. Compared with DM group, in EtOH+DM group, SOD activity, ALDH2 mRNA, and protein expressions were increased; LW/BW and MDA content were decreased. The structures of lung tissue and lamellar bodies were collapsed in DM group; the injury was attenuated in EtOH+DM group. Our findings suggested that, in diabetic rat, pulmonary ALDH2 expression was decreased accompanying lung injury. EtOH at low concentration decreased diabetes induced lung injury through activating ALDH2 expression.

## 1. Introduction

According to an estimation of the World Health Organization (WHO), more than 366 million people worldwide will have diabetes mellitus in 2030; the urban population in developing countries is projected to double between 2000 and 2030. Diabetes mellitus (DM) is an endocrine and metabolic disease which affects almost all organs in our body. Although the lungs are not thought to be primarily affected by diabetes and lung physiological and structural abnormalities are happening in both type 1 and type 2 diabetes, the lung should be considered as a “target organ” [1].

Chronic hyperglycemia is considered as the main reason for diabetes complications. In the process of prolonged increased glucose concentration, reactive oxygen species (ROS) overproduction may be one of the key factors which induce organ injury. Decreased antioxidative function and increased oxidative stress were seen in the lung of diabetic rats and rabbits [2, 3]. Mitochondria dysfunction will cause oxidative stress injury which is one of the underlying factors for neurodegenerative diseases, diabetes, cardiovascular diseases, cancer, and so on [4]. Physiological hypoxia triggers the changes of mitochondrial redox and increases superoxide generation at Complex III in alveolar epithelial cells [5]. Mitochondrial aldehyde dehydrogenase 2 (ALDH2) is a nuclear-encoded mitochondrial enzyme that localizes in mitochondrial matrix [6]; ALDH2 protein had been detected in liver, lung, heart, kidney, testis, esophagus, stomach, colon, and pancreas [7]. Our previous study had reported that, in diabetic rats, ethanol (EtOH) at low concentration offered myocardial protection through activation of ALDH2 expression [8], and upregulation of ALDH2 also plays the protective effect in myocardial ischemia and reperfusion injury, diabetes cardiomyopathy, and kidney and brain injury [9–12]; meanwhile, some researchers reported the association between ALDH2 and lung disease. Xu et al. reported that, in neonatal rat lung, after prolonged hyperoxic exposure, ALDH2 was downregulated. In lung epithelial cells, overexpression of ALDH2 attenuated hyperoxia-induced cell death through reduction of ROS, activation of ERK/MAPK, and PI3K-Akt signaling pathways [13]. But it remains unknown whether activation of ALDH2 expression can prevent diabetes induced lung injury. So in this study, we mimic diabetes model by intraperitoneal injection of streptozotocin (STZ) to observe the role of EtOH at low concentration in diabetes induced lung injury and analyze the related mechanisms.

## 2. Materials and Methods

### 2.1. Animals

Male Sprague-Dawley (SD) rats (200–250 g) obtained from the Animal Center of Bengbu Medical College were selected for the study. All animal studies were approved by the Animal Ethics Committee of Bengbu Medical College and performed in accordance with the ethical standards.

### 2.2. Chemicals and Reagents

**Figure 1:**
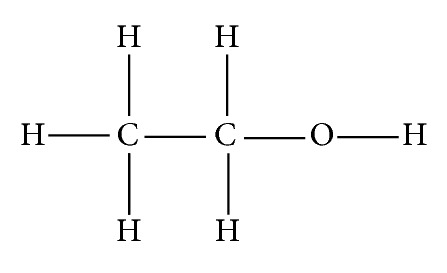
The biochemistry structure of ethanol.

Streptozotocin (STZ) was purchased from Sigma (St. Louis, MO, USA). Malondialdehyde (MDA) and superoxide dismutase (SOD) assay kits were from Nanjing Jiancheng Bioengineering Institute, China. Ethanol (EtOH, Figure 1) was purchased from Bengbu New Chemical Reagent Factory, China. The primers used were as follows: for ALDH2 forward: 5-GTG TTC GGA GAC GTC AAA GA-3′ and reverse: 5′-GCA GAG CTT GGG ACA GGT AA-3′ and the product size was 187 bp; for *β*-actin forward: 5′-GAT GGT GGG TAT GGG TCA GAA GGA C-3′ and reverse: 5′-GCT CAT TGC CGA TAG TGA TGA CT-3′ and the product size was 630 bp. Mouse anti-ALDH2 and anti-*β*-actin monoclonal antibodies were from Santa Cruz Biotechnology (CA). Goat anti-mouse secondary antibodies were purchased from Boston Co., Ltd., Wuhan, China.

### 2.3. Induction of Diabetes and Experimental Protocol

STZ at 55 mg/kg freshly dissolved in 0.1 mol/L sodium citrate buffer (pH 4.5) was injected intraperitoneally to induce diabetic models in overnight fasted rats. The rats in control group were injected with a similar volume of sodium citrate buffer alone. The rats with plasma fasting blood glucose level higher than 16.7 mmol/L 72 h after injection were regarded as diabetic [8]. All rats were fed for eight weeks. Animals were randomly divided into control, diabetes (DM), and EtOH+DM groups, respectively (*n* = 6). In EtOH+DM group, DM rats were fed with 2.5% EtOH in their drinking water for one week to initiate drinking and then it was changed to 5% EtOH continuous access through the 8 weeks.

### 2.4. Detection of Fasting Blood Glucose (FBG) Level, Body Weight (BW), and Lung Weight (LW)

The plasma FBG level and body weight (BW) were measured at the 8th week. Lung weight (LW) was determined after the rat was sacrificed. The ratio of LW/BW was calculated.

### 2.5. Detection of MDA Content and SOD Activity in Lung Tissue

At the end of the experimental period, 0.1 g lung tissue was homogenized in ice-cold PBS buffer. The supernatant was collected after centrifugation for 20 min (2000 rpm). The protein concentration was measured by the Bradford method. Malondialdehyde (MDA) content and superoxide dismutase (SOD) activity were detected according to the instruction manual.

### 2.6. Histological Observation by HE Staining

For histological analysis by light microscope, the lung tissue was harvested and fixed in 4% paraformaldehyde for 24 hours, embedded in paraffin and cut into 5 *μ*m thick serial sections, and then stained with hematoxylin and eosin (HE) for light microscope observation. Lung injury degree was evaluated according to Mikawa's scoring standards: (1) alveolar congestion, (2) hemorrhage, (3) infiltration or aggregation of neutrophils in airspace or vessel wall, and (4) thickness of alveolar wall/hyaline membrane formation. Each item was scored on a 5-point scale as follows: 0: minimal damage, 1: mild damage, 2: moderate damage, 3: severe damage, and 4: maximal damage. The final lung injury score was the summation of the four items [14].

### 2.7. Ultrastructure Observation of Type II Alveolar Cell by Transmission Electron Microscope

Lung tissue was dissected and small pieces were fixed with 2.5% glutaraldehyde in 0.1 mol/L cacodylate buffer for 2 h and postfixed in 1% osmium tetroxide in 0.1 mol/L cacodylate buffer for 1 h. Ultrathin sections were cut and contrasted with uranyl acetate followed by lead citrate and observed with JEM-1230 transmission electron microscope (JEOL, Japan). The changes of Type II alveolar cell were observed.

### 2.8. Detection of Pulmonary ALDH2 mRNA by RT-PCR

The expression of pulmonary ALDH2 mRNA was detected by RT-PCR. Briefly, total RNA was extracted with TRIzol according to the manufacturer's instructions. A total RNA (2 mg) were reverse transcribed to cDNA, and PCR was performed by a routine method. PCR products were analyzed on 1% agarose gel. Quantification of the result was determined through measuring the optical density of the labeled bands; the value was normalized to *β*-actin intensity level.

### 2.9. Detection of Pulmonary ALDH2 Protein Expression by Western Blot

The pulmonary ALDH2 protein expression was detected by western blot [8]. Anti-ALDH2 (1 : 500) antibody was used. Mouse anti-*β*-actin antibody (1 : 500) was used as an internal control. The immunoblots were exposed to X-ray film and analyzed with a digital image system.

### 2.10. Statistical Analysis

All results were evaluated as mean ± S.E.M. Statistical comparisons were carried out by one-way variance analysis and the Newman-Keuls test. *P* < 0.05 was considered as statistically significant.

## 3. Results

### 3.1. Changes of Fasting Blood Glucose Level

In contrast to control group, FBG levels in DM and EtOH+DM groups were increased significantly and FBG level in EtOH+DM group was lower than in DM group (Table 1).

### 3.2. Changes of Body Weight, Lung Weight, and the Ratio of Lung Weight to Body Weight

Compared with control animals, body weight (BW) and lung weight (HW) were significantly decreased in DM and EtOH+DM groups, and LW/BW was increased in DM group. In contrast to DM group, BW was increased and LW/BW was decreased in EtOH+DM group (Table 1).

### 3.3. Changes of SOD and MDA in Lung Tissues

In contrast to control rats, pulmonary SOD activity was decreased in DM group while MDA content was increased in DM and EtOH+DM groups. In EtOH+DM group, SOD activity was higher while MDA content was lower than in DM group (Table 2).

### 3.4. The Histological Changes of Lung Tissue

**Figure 2:**
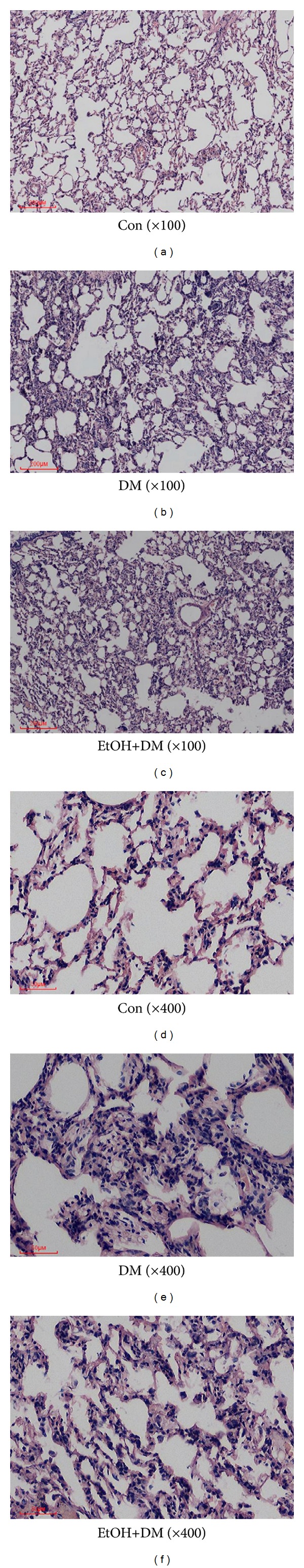
Histological observation of lung tissue in different groups.

In control group, alveolar walls in lung tissue had unique shape and size, and hemorrhage and inflammatory infiltration were rare. In DM group, alveolar septum was thickened widely, infiltration of inflammatory cells and hemorrhage appeared, and the lung injury score was higher than in control group. The injury was ameliorated in EtOH+DM group than in DM group (Figure 2 and Table 1).

### 3.5. Ultrastructural Changes of Type II Alveolar Cell

**Figure 3:**
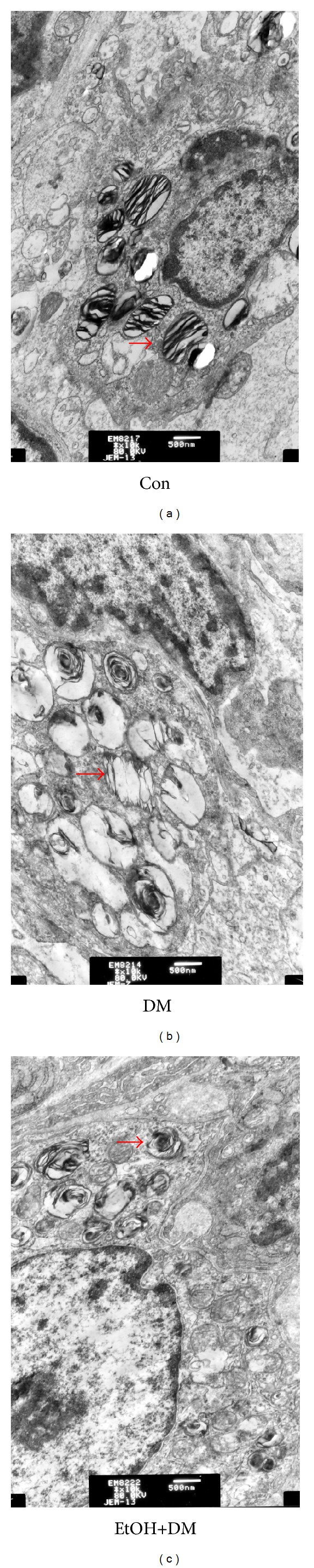
Transmission electron microscopy pictures of Type II alveolar cell in different groups (×15 K).

In control group, many regular structure lamellar bodies appeared in Type II alveolar cell cytoplasm. In DM group, the structure of lamellar bodies was collapsed, discontinuous, and vacuolated. In EtOH+DM group, the injury degree was attenuated (Figure 3).

### 3.6. Changes of ALDH2 mRNA and Protein Levels in Lung Tissue

**Figure 4:**
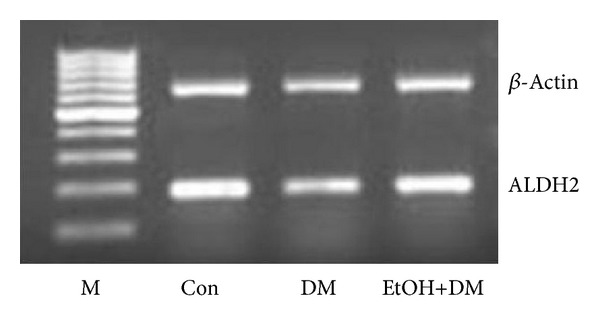
ALDH2 mRNA expression by RT-PCR.

**Figure 5:**
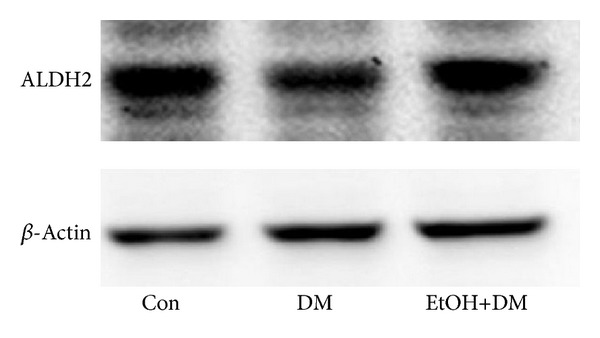
ALDH2 protein expression by western blot.

Compared with control group, the expressions of pulmonary ALDH2 mRNA and protein were decreased in DM group; compared with DM group, pulmonary ALDH2 mRNA and protein expressions were increased in ETOH+DM group (Figures 4 and 5 and Table 3).

## 4. Discussion

In the present study, we observed that lung oxidative stress injury occurred in diabetic rats, which was indicated by the increase of pulmonary MDA content and decrease of SOD activity; meanwhile, the decrease of pulmonary ALDH2 mRNA and protein expressions happened accompanying the happening of lung swelling, the destruction of pulmonary tissue, and Type II alveolar cell structure. When the diabetic rats were treated with EtOH at low concentration which was reported to activate ALDH2 expression [8, 10], lung ALDH2 mRNA and protein expressions were increased, lung oxidative stress injury and swelling degree were attenuated, the destruction of pulmonary tissue and Type II alveolar cell structure was alleviated, suggesting that downregulation of pulmonary ALDH2 was likely to be correlated with oxidative stress injury in diabetic rats, and activation of ALDH2 with EtOH at low concentration attenuated diabetes induced lung injury and oxidative stress overload.

In the development of diabetes, oxidative stress induced by chronic hyperglycemia plays a key role in the pathogenesis of diabetes-related complications including lung diseases. Hyperglycemia can undergo autoxidation and generate oxygen radicals. Diabetes induces impairment of defense system, which is associated with reduced antioxidant capacity, abnormal activity, or expression of antioxidant enzymes [15]. An increase of MDA, a lipid peroxidation marker, accompanied by the depressing of SOD activity (one of the most important endogenous antioxidase enzymes), is assessed to imply oxidant damage. Oxidative stress can destroy the pulmonary structure. In our study, we observed that, in diabetic rat, pulmonary MDA content was increased with the decrease of SOD activity, and the structures of lung tissue and Type II alveolar cell were destroyed, suggesting the unbalance of lung antioxidative system induced lung injury in diabetes rats. When the diabetic rat was treated with EtOH at low concentration, pulmonary MDA content was decreased and SOD activity was increased accompanying the recovery of lung tissue and Type II alveolar cell structures in contrast to diabetic rat, suggesting that EtOH protected the lung tissue through antioxidative stress role.

ALDH2 is an enzyme that detoxifies aldehydes to carboxylic acids. The role of ALDH2 in oxidative stress had been reported by many researchers. Activation of ALDH2 with alda-1 alleviated cardiac ischemic and reperfusion injury with the decrease of 4-HNE in rodent models [8]. ALDH2 overexpression rescued neuronal survival against 4-HNE treatment in PC12 cells [16]. In male C57BL/6 mice, activation of ALDH2 with EtOH prevented renal ischemia and reperfusion injury with suppressed lipid peroxidation and increased SOD activity [12]. ALDH2 prevented ROS-induced vascular contraction in angiotensin-II induced hypertensive mice [17]. But few reports focus on the relationship of pulmonary ALDH2 and diabetes induced lung injury. ALDH2 overexpression attenuated hyperoxia-induced cell death in lung epithelial cells through reduction of ROS, activation of ERK/MAPK, and PI3K-Akt signaling pathways [13]. Since diabetes induced lung injury accompanied with oxidative stress, while increasing ALDH2 expression could decrease oxidative stress, so we investigated whether activating pulmonary ALDH2 expression by EtOH could decrease lung oxidative stress injury in diabetic rats. The results displayed pulmonary ALDH2 mRNA and protein expressions were decreased in diabetic rats, while EtOH treatment increased ALDH2 expression and, meanwhile, decreased oxidative stress injury; it suggested that, in diabetes induced lung injury, the aggravation of oxidative stress may be derived from the decrease of pulmonary ALDH2 expression and improving pulmonary ALDH2 expression could be against the happening of hyperglycemia induced oxidative stress.

It is worthwhile to note that, in our study, we selected EtOH to promote ALDH2 expression because there had been many papers reporting that EtOH at a suitable concentration could activate ALDH2 expression and play the protective effect [8, 10, 12, 18]. But in clinic, EtOH is difficult to apply for patients because of its toxicity and addiction. So selecting an appropriate drug to activate pulmonary ALDH2 expression may be beneficial for diabetic patients who had suffered from lung injury.

In conclusion, our results indicated that, in diabetes induced lung injury in rat model, pulmonary ALDH2 expression was decreased. Treatment with EtOH at low concentration can decrease diabetes induced lung injury through activating ALDH2 expression.

## Figures and Tables

**Table 1:** Changes of fasting blood glucose level (FBG), body weight (BW), lung weight (LW), lung weight/body weight (LW/BW), and lung injury score in different groups.

Group	FBG (mmol/L)	BW (g)	LW (g)	LW/BW (mg/g)	Lung injury score
Con	6.05 ± 1.02	443.53 ± 23.84	2.40 ± 0.43	5.39 ± 1.94	1.33 ± 0.81
DM	32.48 ± 3.20**	179.28 ± 28.46**	1.85 ± 0.38*	10.06 ± 1.79**	9.67 ± 1.21**
EtOH + DM	27.15 ± 6.67^∗∗#^	243.75 ± 11.09^∗∗##^	1.68 ± 0.20**	6.87 ± 0.56^##^	6.33 ± 0.82^∗∗##^

*P < 0.05 and **P < 0.01 compared with Con; ^#^ *P* < 0.05 and ^##^ *P* < 0.01 compared with DM.

**Table 2:** Changes of SOD activity and MDA content in lung tissues in different groups.

Group	SOD activity (U/mg prot.)	MDA content (nmol/prot.)
Con	7.55 ± 1.94	3.93 ± 0.40
DM	4.07 ± 0.81**	5.63 ± 0.51**
EtOH + DM	6.45 ± 2.05^#^	4.67 ± 0.71^∗##^

*P < 0.05 and **P < 0.01 compared with Con; ^#^ *P* < 0.05 and ^##^ *P* < 0.01 compared with DM.

**Table 3:** Changes of ALDH2 mRNA and protein expressions in lung tissues in different groups.

Group	ALDH2/*β*-actin mRNA	ALDH2/*β*-actin protein
Con	0.47 ± 0.05	1.17 ± 0.19
DM	0.35 ± 0.04**	0.85 ± 0.10**
EtOH + DM	0.42 ± 0.06^#^	1.14 ± 0.15^#^

**P < 0.01 compared with Con; ^#^ *P* < 0.05 compared with DM.
